# Gamma-Aminobutyric Acid Increases the Production of Short-Chain Fatty Acids and Decreases pH Values in Mouse Colon

**DOI:** 10.3390/molecules22040653

**Published:** 2017-04-20

**Authors:** Min Xie, Hai-Hong Chen, Shao-Ping Nie, Jun-Yi Yin, Ming-Yong Xie

**Affiliations:** State Key Laboratory of Food Science and Technology, Nanchang University, Nanchang 330047, China; ncuskxiemin@163.com (M.X.); chh0810ncu@163.com (H.-H.C.); nie68@sina.com (S.-P.N.)

**Keywords:** gamma-aminobutyric acid, colon, SCFAs, pH values

## Abstract

Gamma-Aminobutyric acid (GABA) could regulate physiological functions in the gastrointestinal tract. The present study aimed to investigate the effect of GABA on colon health in mice. The female Kunming mice were given GABA at doses of 5, 10, 20 and 40 mg/kg/d for 14 days. Afterwards, the short-chain fatty acids (SCFAs) concentrations, pH values, colon index, colon length and weight of colonic and cecal contents were determined to evaluate the effects of GABA on colon health. The results showed that intake of GABA could increase the concentrations of acetate, propionate, butyrate and total SCFAs in colonic and cecal contents, as well as the weight of colonic and cecal contents. The colon index and length of the 40 mg/kg/d GABA-treated group were significantly higher than those of the control group (*p* < 0.05). In addition, decrease of pH values in colonic and cecal contents was also observed. These results suggest that GABA may improve colon health.

## 1. Introduction

The colon can maintain the health of the whole body by absorbing water and electrolytes, and salvaging unabsorbed nutrients [1]. It has been shown that short chain fatty acid (SCFA) and pH values play crucial roles in intestinal tract health [2,3,4]. SCFA are metabolic by-products of the gut microbiota fermentation in the colon [5]. This could provide energy for colonic epithelia to maintain normal colonic cell phenotype, stimulate the growth of colorectal cells, and colonic blood flow [2,6]. Acetate, propionate and butyrate are the primary forms of SCFA. Acetate constitutes a majority of the total SCFA in feces (about 60–75%) [7]. Propionate and butyrate could accelerate differentiation and apoptosis of colon cancer cells, and thus protect the colon from carcinogenesis [8]. In addition, the decrease of pH values was also beneficial to colon health by preventing overgrowth of pH-sensitive pathogenic bacteria in colon [6].

Gamma-aminobutyric acid (GABA) is a non-protein amino acid and main inhibitory neurotransmitter in the central nervous system [9,10], which is expressed in the central nervous system and non-neuronal peripheral tissues including the intestine [11]. It has been authenticated as a ‘new resources’ food by the State Food and Drug Administration in China. Several studies have shown that GABA could regulate many intestinal physiological functions, such as intestinal fluid secretion, blood flow and ulceration [12,13]. The GABA or GABA_A_ receptor has been reported to regulate motility in the small intestinal and colon through regulating acetylcholine release from cholinergic neurons [14,15,16]. Krantis et al. [12] found that GABA improved intestinal mucosal activity by regulating intestinal fluid and electrolyte transport due to its presence in myenteric neurons. In addition, Song et al. [17] also suggested that GABA might play an auxiliary role in the polychemotherapy of colon cancer by inhibiting proliferation of the colon cancer cell. The above studies suggested the pivotal role of GABA in protecting intestinal health. However, to the best of our knowledge, there are few reports on the influences of GABA on colonic metabolism and growth status.

In this study, colonic metabolism parameters such as SCFAs production, pH values in colonic and cecal contents and weight of colonic and cecal contents were analyzed. Colonic growth parameters such as colon length and index were measured to describe the growth status of the colon. The results would have potential value for commercial exploration of GABA, especially on colon health.

## 2. Results

### 2.1. Weight and Health Status of Mice

The body mass of mice during the experiment is shown in Table 1. During the experimental period, the body mass of mice rose but there was no significant difference between the GABA-treated groups and the control group (*p* > 0.05). No obvious abnormal behavior, treatment-related illness or death was observed. The appearance of diarrhea and constipation in mice did not occur. There were also no remarkable differences in hair luster of the mice among the five groups.

### 2.2. Colon Index and Length 

**Figure 1:**
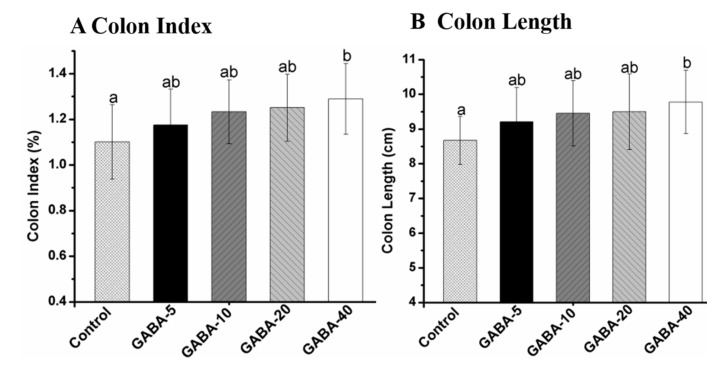
The colon index (**A**) and colon length (**B**) of mice treated with gamma-Aminobutyric acid (GABA). Results are expressed as mean value ± SD (*n* = 12). Data with different letters in the same column means significant difference among groups (*p* < 0.05). Colon index was calculated by using Equation (1).

The effects of GABA on colon index and length are presented in Figure 1. The colon index and length in the 40 mg/kg/d GABA-treated group were significantly increased compared with the control group (*p* < 0.05). However, there was no significant difference between the other GABA groups and the control group (*p* > 0.05). The increments of the colon index and length were in a dose-dependent manner in GABA-treated groups.

### 2.3. Effect of GABA on Weight of Colonic and Cecal Contents

**Figure 2:**
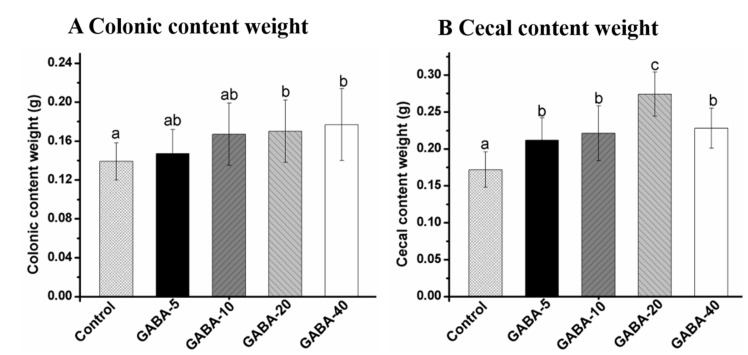
The weight of colonic (**A**) and cecal (**B**) contents of mice treated with GABA. Result are expressed as means value ± SD (*n* = 12). Data with different letters in the same column means significant difference among groups (*p* < 0.05).

The effects of GABA on weight of colonic and cecal contents are presented in Figure 2. Compared with the control group, the colonic content weight in the 20 and 40 mg/kg/d GABA-treated groups were significantly increased (*p* < 0.05). GABA pretreatment significantly increased the cecal content weight compared with the control group (*p* < 0.05). Moreover, the cecal content weight reached the highest value (0.274 ± 0.030) in the 20 mg/kg/d GABA-treated group. Furthermore, the colonic content weight was found to be increased in a dose-dependent manner in the GABA-treated groups.

### 2.4. Effect of GABA on pH Value in the Colonic and Cecal Contents

**Figure 3:**
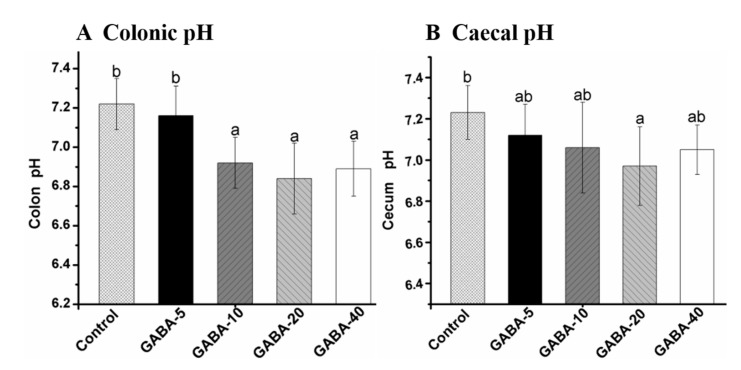
pH values change in colonic (**A**) and cecal contents (**B**) of mice treated with GABA. The results were expressed as mean ± SD (*n* = 12), and evaluated by one way ANOVA with turkey test. Values with different letters indicated significant different among groups (*p* < 0.05).

As one of the key parameters for determining intestinal health, pH values could regulate the growth of microorganisms and enzyme activities in metabolism [18]. The effects of GABA on pH values in colonic and cecal contents are presented at Figure 3A,B respectively. Administration of GABA at doses of 10, 20 and 40 mg/kg/d significantly decreased the pH values (6.92 ± 0.13, 6.84 ± 0.18 and 6.89 ± 0.14) in the colonic content compared with the control group (*p* < 0.05). The pH values (6.97 ± 0.19) in cecal content from the 20 mg/kg/d GABA-treated group were significantly lower than that in the control group (7.23 ± 0.13). Nevertheless, there was no significant difference in pH values between the other GABA groups and the control group in cecal content (*p* > 0.05).

### 2.5. Effect of GABA on Total SCFA in the Colonic and Cecal Contents

**Figure 4:**
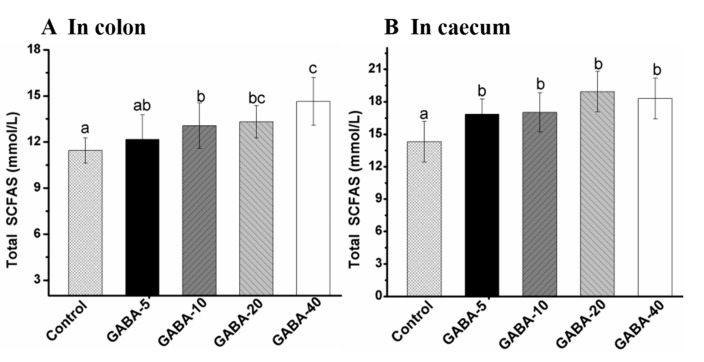
The effect of GABA on total short-chain fatty acid (SCFA) concentrations in the mice colonic (**A**) and cecal (**B**) contents. Data was represented as mean ± SD (*n* = 12), and evaluated by one way ANOVA with turkey test. Values with different letters expressed significant differences among groups (*p* < 0.05).

SCFAs derived from carbohydrate fermentation by anaerobic bacteria are the principal energy source for colonic epithelial cells [19,20,21,22]. The accumulations of SCFAs primarily take place in the proximal colon and cecum in mice [23,24]. Total SCFA concentrations in the colonic and cecal contents are presented in Figure 4A,B. The calibration curves for SCFAs data were linear with all *R*^2^ > 0.999. The significant higher concentrations of total SCFA in the colonic and cecal contents of GABA-treated groups were observed compared with the control group, except for the 5 mg/kg/d GABA-treated group in the colon (*p* < 0.05). The concentration of total SCFA in cecal content was the highest in the 20 mg/kg/d GABA-treated group. The total SCFA concentrations in the colonic content were found to be increased in a dose-dependent manner in GABA-treated groups.

### 2.6. Effect of GABA on Individual SCFA in the Colonic Contents

**Figure 5:**
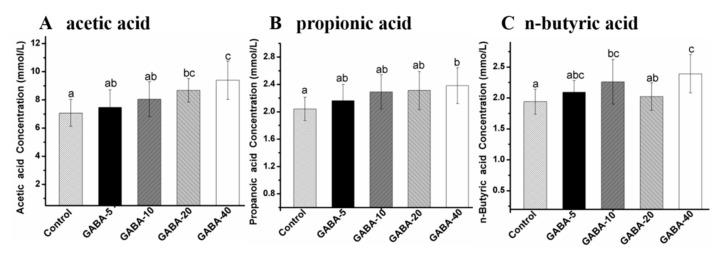

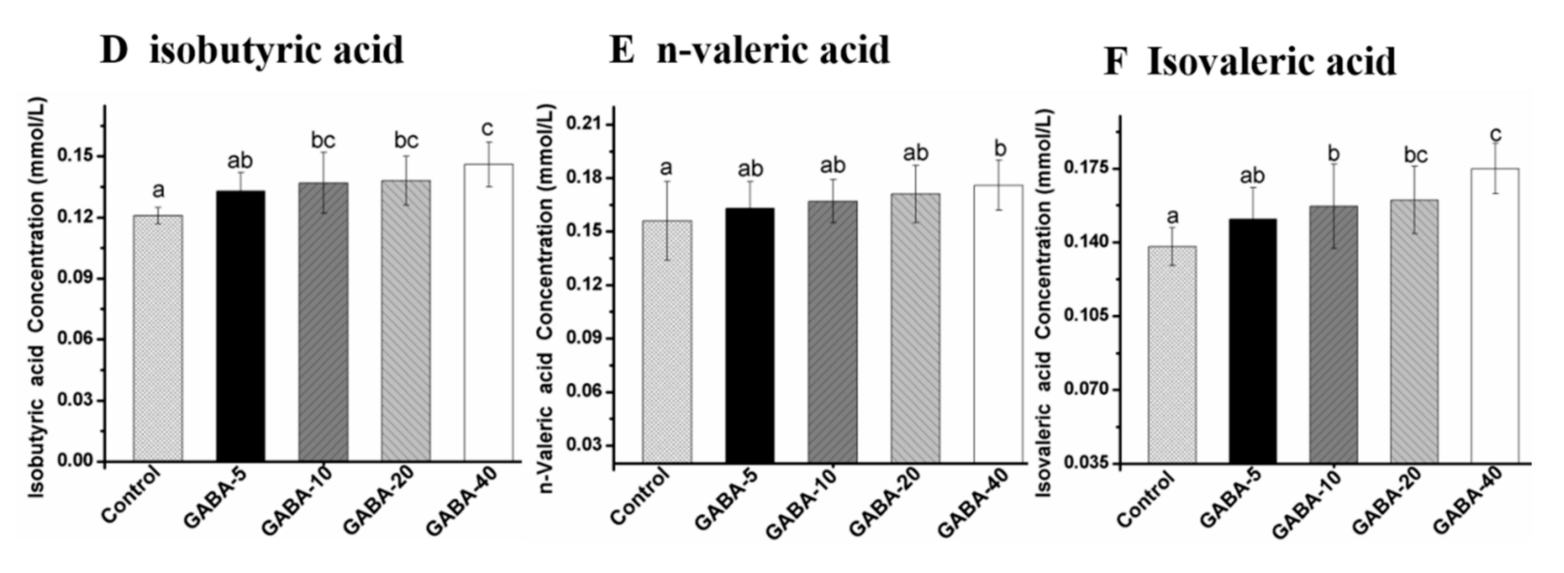
The effect of GABA on individual SCFA concentration in the mice colonic content, acetic acid (**A**), propionic acid (**B**), *n*-butyric acid (**C**), isobutyric acid (**D**), *n*-valeric acid (**E**), isovaleric acid (**F**), respectively The data was presented as mean ± SD (*n* = 12), and evaluated by one way ANOVA with turkey test. Values with different letters in the same chart indicated significant differences among groups (*p*< 0.05).

Acetic acid, propionic, and *n*-butyric acids are the three primary types of SCFAs with an average ratio of 57:22:21 in the large intestine [25]. In contrast, low amounts of isobutyric acid, *n*-valeric acid and isovaleric acid are detected in colonic and cecal contents. The levels of individual SCFA in colonic content are shown in Figure 5. As shown in Figure 5, the significant higher levels of acetic acid, propionic acid, *n*-valeric acid, isobutyric acid, *n*-valeric acid, and isovaleric acid were observed in 40 mg/kg/d GABA-treated group compared with the control group (*p* < 0.05). Moreover, the level of acetic acid in 20 mg/kg/d GABA-treated group and the level of *n*-valeric acid in the 10 mg/kg/d GABA-treated group were significantly higher than those in the control group (*p* < 0.05). Moreover, the levels of isobutyric acid and isovaleric acid in the 10, 20 mg/kg/d GABA-treated groups were also significantly increased compared with the control group (*p* < 0.05). The levels of acetic acid, propionic acid, isobutyric acid, *n*-valeric acid and isovaleric acid were increased in a dose-dependent manner except for the *n*-butyric acid in the colonic content of the GABA-treated groups.

### 2.7. Effect of GABA on Individual SCFA in Cecal Contents

**Figure 6:**
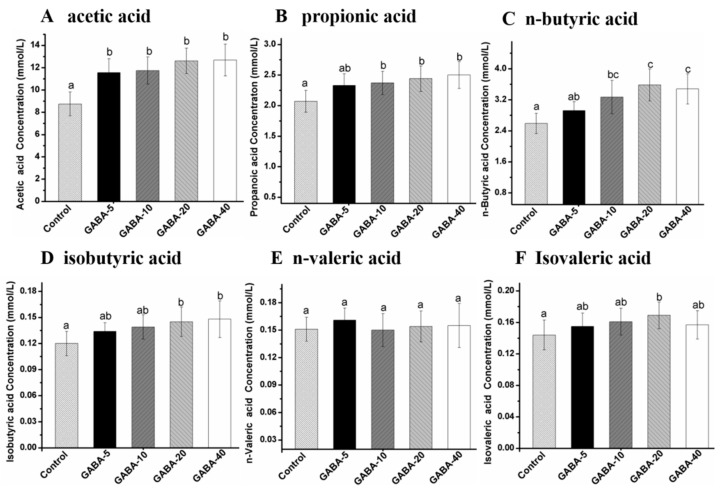
The effect of GABA on individual SCFA in the mice cecal content, acetic acid (**A**), propionic acid (**B**), *n*-butyric acid (**C**), isobutyric acid (**D**), *n*-valeric acid (**E**), isovaleric acid (**F**), respectively. Data was expressed as mean ± SD (*n* = 12), and evaluated by one way ANOVA with turkey test. Results with different letters showed significant differences from each group (*p* < 0.05).

The concentrations of individual SCFA in cecal content are presented at Figure 6. As seen in Figure 6, compared with the control group, administration of GABA significantly increased the concentration of acetic acid in cecal content (*p* < 0.05). The concentrations of propionic acid and *n*-butyric acid in 10, 20 and 40 mg/kg/d GABA-treated groups were significantly elevated compared with the control group (*p* < 0.05). The concentrations of isobutyric acid in 20 and 40 mg/kg/d GABA were significantly higher than those in the control group (*p* < 0.05). In addition, the concentrations of *n*-valeric acid and isovaleric acid were increased, with no significant difference (*p* > 0.05). The concentrations of acetic acid, propionic acid and isobutyric acid were augmented with a dose-dependent manner in GABA-treated groups.

## 3. Discussion

The aim of present study was to evaluate the effect of GABA on colon health. Previous studies have suggested that the colon health was closely correlated to the increase of SCFAs production, colon length and index, and the decrease of pH values, which provided the theoretical basis for our research [26,27]. Our results, for the first time, suggest that GABA may promote colon health by increasing the production of SCFAs, colon index, colon length, weight of colonic and cecal contents and reducing the pH values in mice.

SCFAs have been reported to contribute to colon heath through many physiological mechanisms, which included inhibiting the growth of the intestinal pathogenic bacteria [28,29], and reducing inflammatory responses [30] and secondary bile acid formation in the colon [27]. Furthermore, the increase of SCFAs concentrations could result in increasing mineral substance absorption and stimulating epithelial cell proliferation [31]. Acetate, as the most abundant SCFA in colonic and cecal contents, has a trophic effect on colonic epithelium by increasing mucosal blood flow [32]. It has been demonstrated that acetate and propionate could obviously reduce serum cholesterol and inhibit adipogenesis [33,34,35]. Butyrate could be absorbed by the colonocyte as the primary energy source for the colonic epithelium to boost the growth of colonic tissue and colonic mucosa [27,36]. It could also maintain gut health through regulating intestinal motility, reducing oxidative stress, enhancing immune activity and inhibiting colon cancer [37,38,39]. Additionally, some literatures have shown that the increase of SCFAs may beneficial to maintain colon health [26,40]. In our study, the concentrations of total SCFAs, acetic acid, propionic acid and butyric acid in colonic and cecal contents of GABA-treated groups were higher than those in the control group. Therefore, we suggest that increasing SCFAs concentrations is a critical factor for GABA to improve colon health. However, the mechanism of the GABA effect on increase of SCFAs is still unclear. GABA has been established as a possible route for gut microbiota to communicate with the gut-brain-axis, produced and utilized by intestinal probiotic bacteria, which may potentially be used as a “prebiotic” for intestine health [41,42,43]. Whether GABA could increase SCFAs by its action on gut microbiota needs further investigation in the future.

Interestingly, the results in our study also showed that pretreatment with GABA increased the concentrations of isobutyrate and iso-valerate in line with the increase of SCFA production, which is in accordance with the results of several studies in the literature [44,45,46]. In the large intestine, protein is fermented by bacteria, which produces SCFAs, branched-chain fatty acids (BCFA) such as isobutyrate and iso-valerate, and potentially toxic compounds [47]. Additionally, it was well known that the basal diet contained proteins in our study. GABA could increase food intake by stimulating ingestive behavior [48], which may result in the increases of isobutyrate and iso-valerate concentrations.

It is worth noting that the increase of colon length and index may be involved in promoting colon health [26,44]. A greater-than-normal colon length could promote colon health by preventing degenerative bowel disease [49]. Moreover, the colon length was decreased in the colitis induced by a relatively low dose of dextran sulfate sodium [50]. Campbell et al. [3] found that the increase of colonic and cecal tissue weight might result from the augmentation of crypt depth and cell density by providing butyrate as an energy source. The results in this study indicated that pretreatment with GABA could increase colon length and index compared with the control group. Moreover, GABA has been reported to protect intestinal mucosal by increasing crypt depth, intestinal wall thickness and the number of goblet cells [51], which may account for the increase of colon index and length in our study. 

The weights of colonic and cecal contents were involved in improving intestinal health [52,53]. Zhou et al. [40] reported that the increase of the cecal content and cecal wall weight might result from the formation of SCFAs. Our results indicated that GABA pretreatment at doses of 20 and 40 mg/kg/d could significantly increase the weight of colonic and cecal contents, compared with the control group.

pH values in colonic and cecal contents is also an important indicator to evaluate the health status of the colon. Lower colonic pH has been suggested to inhibit chronic bowel diseases, colorectal cancer, and the bacterial conversion of primary to secondary bile acid [54]. A lowering pH value could also prevent the growth of pathogenic bacteria such as Salmonella [55,56]. Our results indicated that the pH values were decreased in colonic and cecal contents in GABA-treated groups, which was in line with these studies. Furthermore, it has been reported that the increase in SCFAs productions could contribute to the decrease of luminal pH [18,27]. This implies that GABA may lower pH values due to the increase of SCFAs production.

GABA plays a crucial role in colon health in mice. However, whether dietary GABA could reach the cecum and colon in order to work is still unclear. It has been shown that GABA exerts its actions through three types of receptors, including GABA_A_, GABA_B_ and GABA_C_ receptors [57]. These GABA receptors could be found to express in the colon [57,58]. In addition, GABA could modulate peristaltic activity in mouse colon through activation of GABA_A_ or GABA_B_ receptors [16]. The GABA_A_ receptor was also reported to increase the sodium and water intake by the disinhibition effect of GABAergic [59]. Furthermore, the colon health was closely related to sodium and water absorption [60]. It has also been shown that the increase of sodium and water absorption contributed to SCFA production and absorption [61,62]. Therefore, the effect of GABA on colon health may be associated with sodium and water absorption regulated by GABA receptors.

## 4. Materials and Methods

### 4.1. Materials

GABA (≥99% purity) was purchased from Solarbio Company (Beijing, China), the high-purity SCFAs were used as standard solutions for gas chromatographic analysis. Acetic acid (100% purity) was obtained from Merck Co. (Darmstadt, Germany). Propionic acid (100% purity) was purchased from Janssen Chimica (Beerse, Belgium). Isobutyric acid (99.9% purity), *n*-butyric acid (100% purity), *n*-valeric acid (99.9% purity) and isovaleric acid (100% purity) were from Sigma Corporation (St. Louis, MO, USA). All the others were of analytical grade.

### 4.2. Animals

All experiments were approved by the Animal Care Review Committee (Animal application approval number 0064257), Nanchang University, China. Female 6-week-old Kunming mice, weighing 18.0 ± 2.0 g, were provided by Animal Breeding Center, Nanchang University, Nanchang, Jiangxi Province, China. All animals were cared for in accordance with the Guide for Care and Use of Laboratory Animals, published by the Institute of Laboratory Animal Resources, Commission on Life Sciences, National Research Council, National Academy Press (NIH Publication 85–23, revised 1996).

### 4.3. Animal Experiment

All mice were acclimatized at least 7 days before the experiments and fed with the same basal diet from Animal Breeding Center, Nanchang University, Jiangxi Province, China. The composition of the basal diet was in accordance with the report of Hu et al. [63]. Sixty mice were randomly divided into 5 groups, including the 5, 10, 20 and 40 mg/kg/d GABA groups (GABA-5, GABA-10, GABA-20 and GABA-40) and the control group. The doses for the GABA administrated were selected according to the standard issued by the National Health and Family Planning Commission of the People’s Republic of China, where GABA consumption for people is less than 500 mg per person per day.

All mice were raised in a room with controlled temperature at 25 ± 0.5 °C, relative humidity of 50% ± 5% and a 12 h light/dark cycle. The mice in the GABA-treated groups were given different doses of GABA. The mice in the control group were given the corresponding volume of saline. Furthermore, all mice were weighed at approximately 09:00 every day before oral administration, and the volume of GABA and saline was adjusted in a dose of 10 mL/kg according to the body mass. All mice were killed after 14 days of oral administration. The colon and cecum were aseptically removed immediately, placed on an ice-cold plate and opened longitudinally. The colonic and cecal contents were collected, weighted and stored at −80 °C for determining SCFAs contents and pH values. The colon tissue was rinsed with physiological saline, and collected for the measurements of colon index.

### 4.4. Analysis of SCFAs

The colonic and cecal contents were weighed and immediately placed into a round-bottomed stoppered tube in an ice-cold water bath. The colonic and cecal contents were diluted by adding deionized water in a ratio of 1:7. All the samples were mixed for 3 min by a vortex mixer and executed with ultrasonic-processing continuously for 5 min. The processes of the vortex and ultrasound were repeated once. The sample was extracted by standing in an ice-cold water bath for 20 min and then centrifuged at 4800× *g* for 20 min at 4 °C. The supernatant was transferred into another tube. All the above processes were repeated once. The supernatant was mixed and divided into two parts for analysis of SCFAs by gas chromatography and determination of pH values.

Gas chromatography was executed using an Agilent 6890 N GC system equipped with a flame ionization detector and an N10149 automatic liquid sampler (Agilent Technologies Inc., Palo Alto, CA, USA). The running parameters were set and adjusted according to the report of Hu et al. [63]. Standard curves were made in the range 1–20 mmol/L for acetic acid, 0.5–12.5 mmol/L for *n*-butyric acid, 0.75–15 mmol/L for propionic acid, 0.05–1.25 mmol/L for isobutyric acid, *n*-valeric acid and isovaleric acid (3 replicated for each level, 8 concentration levels), with deionized water as a blank control. The gas chromatography conditions were shown in Table 2.

### 4.5. Determination of Colon Index and Length 

The colon length was measured by the ruler. Then, the colonic contents and fat around the colon were cleared away completely. The colon tissue was weighed. The colon index was calculated by the following formula [26,44]:

(1) Colon index (%) = W_1_/W_2_ × 100,

where W_1_ represented the weight of colonic tissue of every mouse (g), W_2_ represented the body weight of the mice (g).

### 4.6. Determination of pH Value

Another portion of the supernatant (Collected in Section 2.4) was used to determine pH values by a PB-21 pH meter (Sartorius Corporation, Gottingen, Germany). The same sample was repeatedly measured 3 times for pH values.

### 4.7. Statistical Analysis

Statistical analysis was carried out by using SPSS Statistics Software (version 19.0, Chicago, IL, USA). The results were expressed as mean ± standard deviations (SD) with 12 animals in each group (*n* = 12). Data was evaluated by one-way analysis of variance, followed by Tukey test to calculate the significant difference. The significance level was set at *p* < 0.05.

## 5. Conclusions

In our study, administration with GABA increased the productions of SCFAs, colon index, colon length, weight of colonic and cecal contents and lowered the pH value in colonic and cecal contents. These findings suggested that GABA may be beneficial in improving colon health. However, the mechanisms by which GABA affect colon health need to be further explored.

## Figures and Tables

**Table 1:** Body mass of mice during the experiment period ^a^ (g).

Group	0 Day	7 Day	14 Day
Control	22.98 ± 1.72 a	27.46 ± 1.77 b	30.74 ± 2.08 c
GABA-5	22.40 ± 1.47 a	27.15 ± 2.57 b	30.68 ± 1.93 c
GABA-10	22.96 ± 1.25 a	27.45 ± 1.62 b	30.71 ± 1.75 c
GABA-20	23.13 ± 1.32 a	28.14 ± 1.51 b	31.62 ± 1.54 c
GABA-40	22.89 ± 2.06 a	27.71 ± 1.89 b	31.11 ± 1.84 c

^a^ Data was expressed as mean ± SD (*n* = 12).Values in the column with different letters indicated significant differences (*p* < 0.05).

**Table 2:** Gas chromatographic conditions.

Condition	Running Parameter
Chromatographic column	HP-INNOWAX (30 m × 0.32 mm × 0.5 μm)
Detector and temperature	FID, 240 °C
Carrier gas and flow rate	N_2_, 1.2 mL/min
Injection volume	0.2 μL
Air velocity	300 mL/min
Hydrogen flow rate	30 mL/min
Split ratio	0
Temperature-rising procedure	100 °C (0.5 min, 4 °C/min) − 170 °C; 170 °C (20 °C/min) − 230 °C
